# The global burden of kidney cancer: trends in mortality and incidence with predictions to 2025

**DOI:** 10.1097/CEJ.0000000000001000

**Published:** 2025-12-16

**Authors:** Silvia Mignozzi, Claudia Santucci, Alberto Briganti, Francesco Montorsi, Carlo La Vecchia, Giovanni Corso, Eva Negri, Giorgio Gandaglia

**Affiliations:** a Department of Clinical Sciences and Community Health, University of Milan; b Fondazione IRCCS Ca’ Granda, Ospedale Maggiore Policlinico; c Unit of Urology/Division of Oncology, Gianfranco Soldera Prostate Cancer Lab, Urological Research Institute, IRCCS San Raffaele Scientific Institute; d Division of Breast Surgery, European Institute of Oncology IRCCS; e Department of Oncology and Hemato-Oncology, University of Milan, Milan; f Department of Medical and Surgical Sciences, University of Bologna, Bologna, Italy

**Keywords:** incidence, kidney cancer, mortality, trends

## Abstract

Over the past three decades, the incidence of kidney cancer has been increasing worldwide. Mortality trends, however, differed over time and across regions, showing downward trends in most high-income countries, but upward trends mainly in Latin America and a few Eastern and Central European countries. We provide updated figures on the epidemiology of kidney cancer worldwide. We analysed mortality data from the WHO in 33 selected countries worldwide from 2000 to 2023 or the latest available year. We computed age-standardised mortality rates (ASMRs) and evaluated temporal trends with joinpoint regression models. We predicted mortality figures for 2025, using a logarithmic Poisson count data joinpoint regression model. We analysed incidence from Cancer Incidence in Five Continents Volume XII. In 2020, the highest male ASMRs (5/6 per 100 000) were in Slovakia, the Czech Republic, Hungary, and Argentina. Female rates remained below 2/100 000, except in the Czech Republic and Hungary. Mortality declined for both sexes in most countries, except for Latin America. Predictions to 2025 suggest that kidney cancer mortality is expected to decline for both sexes in approximately all countries. The incidence of kidney cancer showed generalised upward trends. We observed differing trends between kidney cancer incidence and mortality. Kidney cancer mortality declined in most countries, likely reflecting reductions in smoking mainly among males, improvements in diagnosis and treatment, and better hypertension control. Incidence of kidney cancer has been rising in most high-income countries, likely reflecting widespread increases in the use of imaging techniques.

## Introduction

Over the last three decades, kidney cancer incidence has increased (Cirillo *et al*., 2024). This is likely because of the widespread adoption of diagnostic procedures (Bahadoram *et al*., 2022), particularly abdominal ultrasound, which has contributed to the incidental diagnosis of indolent small asymptomatic renal masses that would otherwise go unnoticed. In addition, this could be attributable to an increasing prevalence of established risk factors, such as overweight and obesity (Boutari and Mantzoros, 2022). Mortality trends varied by region and time, with favourable trends in Northern and Western Europe, the North America, Israel, Japan, and Australia since the 1990s. Mortality has been increasing in Latin America, particularly Brazil, but also in Southern and Eastern Europe (Cirillo *et al*., 2024). The 5-year overall survival rate varied from 40 to 75%, depending on the geographic area (Larcher *et al*., 2025). A correct comparison and understanding of the trends in kidney cancer incidence and mortality is of importance for patient counselling, the development of prevention strategies, the implementation of early detection programs, and to inform healthcare planning and resource planning and allocation at both national and global levels; however, available studies are often hampered by limitations in data quality, which might considerably vary in terms of completeness, accuracy, and frequency of updates across different countries and regions.

To provide updated information, we analysed trends in mortality and incidence from kidney cancers (excluding tumours from the renal pelvis) across a subset of selected countries worldwide. We also aim to provide predictions of kidney cancer mortality rates for the year 2025.

## Materials and methods

### Mortality

We extracted official mortality data from kidney cancer and other urinary sites from the WHO database (World Health Organization Statistical Information System, 2025), for selected countries worldwide, according to mortality coverage (≥90%), population size (for European countries over 5 000 000 inhabitants while for the other countries worldwide over 20 000 000), and high data quality (WHO, 2020). We also analysed the 27 countries that are current European Union (EU) member states as a whole, defined as the EU-27. For each country, we considered the number of certified deaths from kidney cancer (excluding renal pelvis) from 2000 to 2023 or the most recent available year, coded as C64 in the 10^th^ Revision of the International Classification of Diseases or as 189.0 in the 9^th^ Revision, according to the availability of each country. Mortality data for the year 2000 are missing for the UK, and data for 2020–2021 are missing for Portugal. The corresponding resident population data were extracted from the United Nations (UN) Population Division database (United Nations, Department of Economic and Social Affairs, Population Division, 2017). For each country, calendar year, and sex, we calculated 5-year age-specific rates and the age-standardised mortality rate (ASMRs) per 100 000 person-years, using the world standard population, at all ages and for the age groups 25–49, 50–69, and over 70 years. We estimated the ASMRs for the quinquennium 2010–2014 and the year 2020 with the corresponding percent change in rates.

For the EU-27 and the most populous (≥10 million inhabitants) countries, we fitted a joinpoint regression model from 2000 until the most recent available year (Kim *et al*., 2000, 2022). We thus identified the joinpoint(s), that is, the year(s) when a change in the linear slope (on a log scale) of the temporal trend occurred, by testing up to a maximum of five joinpoints. As a summary measure, we also estimated the average annual percent change (AAPC) over the entire study period.

For the largest countries (≥20 million), we fitted a Poisson joinpoint regression model to the number of deaths in each 5-year age group and run a linear regression over the most recent trend segment identified by the joinpoint model to obtain the estimated number of deaths for each age group for 2025. We extracted predicted population data from the UN database (United Nations, Department of Economic and Social Affairs, Population Division, 2017). Thus, we computed predicted ASMRs and 95% prediction intervals using the matrices of predicted age-specific death counts and predicted populations.

### Incidence

We obtained incidence data until 2017 from the Cancer Incidence in Five Continents database, Volume XII (Bray *et al*., 2023). We considered countries included in the mortality analyses with over 10 million inhabitants that also provided incidence data. For countries with more than one cancer registry, we aggregated data to ensure the largest geographic coverage, and we restricted analyses to the longest common calendar period between registries. For each country, we estimated the age-adjusted incidence rates (ASIR) for the periods 2005–2007 and 2015–2017 with the corresponding percent change in rates. In addition, we examined trends from 2000 to 2017 using locally estimated scatterplot smoothing (LOESS) regression (span: 0.3).

For data analyses, we used the software R version 4.3.2; (R Development Core Team, 2024) and SAS version 9.4 (SAS Institute Inc., Cary, North Carolina, USA).

## Results

**Fig. 1:**
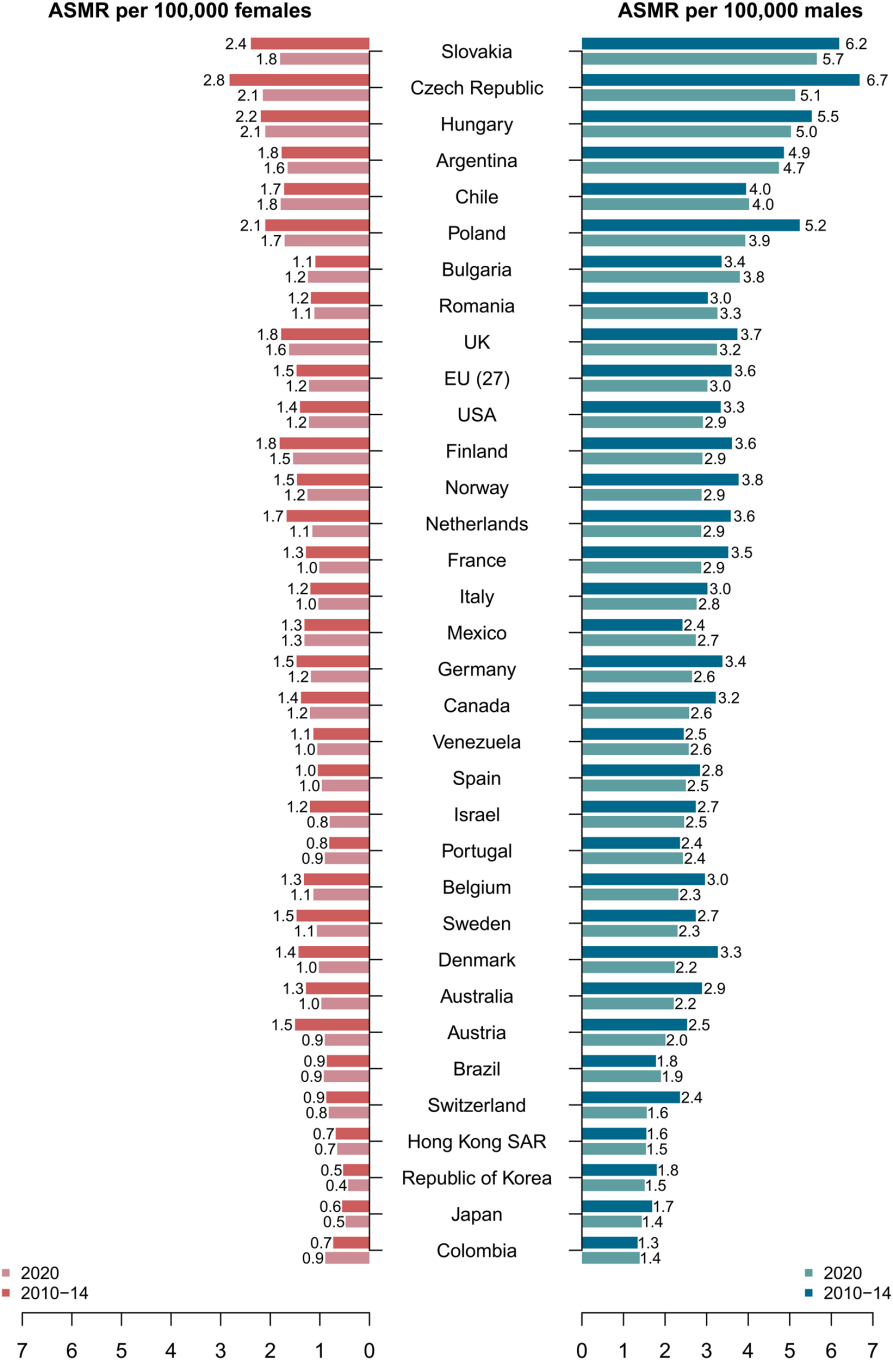
Bar plots of ASMR per 100 000 from kidney cancer for the periods 2010–2014 and 2020 in males and females separately in selected countries worldwide, ordered from the highest to the lowest 2020 male rate. ASMR, age-standardised mortality rate.

**Fig. 2:**
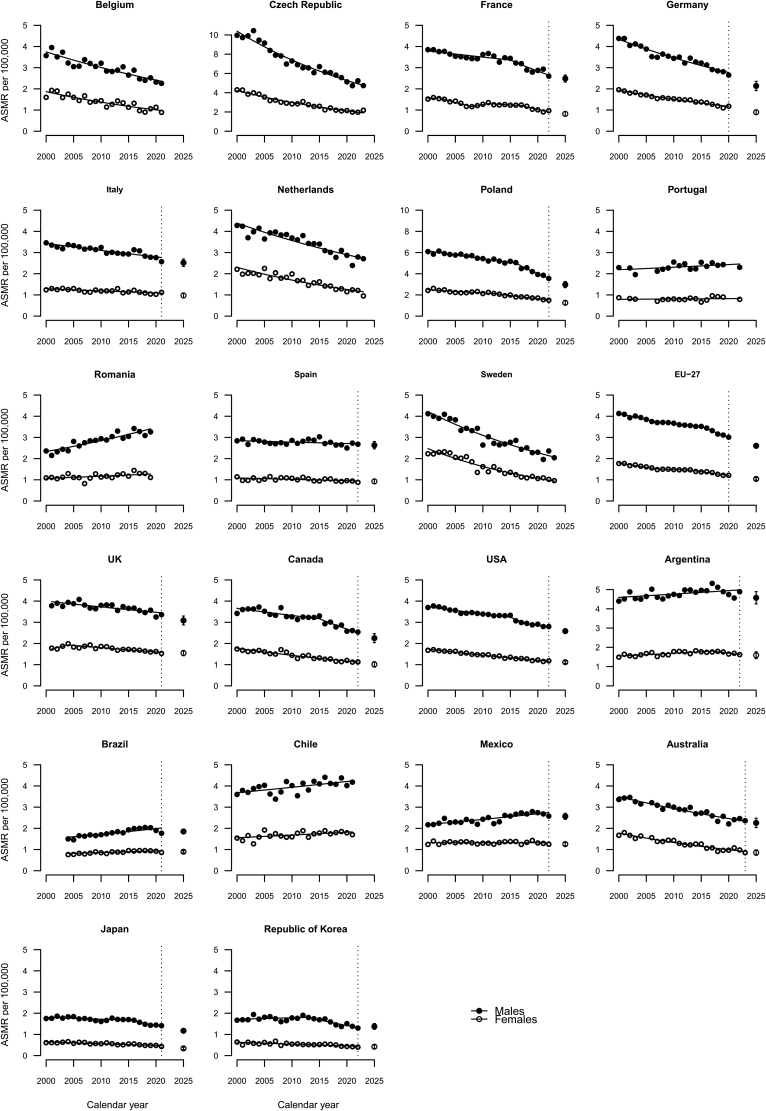
Annual ASMR per 100 000 males and females at all ages from kidney cancer in major countries worldwide and the EU-27, the resulting joinpoint regression models and predicted rates (for countries with ≥20 million inhabitants and the EU-27) for the year 2025 with 95% prediction intervals. ASMR, age-standardised mortality rate.

Table 1 gives the ASMRs per 100 000 males and females at all ages in selected countries worldwide for the periods 2010–2014 and 2020. Among males, the highest ASMRs in 2020 were recorded in Slovakia (5.65/100 000), followed by the Czech Republic (5.13/100 000), Hungary (5.03/100 000), Argentina (4.74/100 000), and Chile (4.02/100 000). Australasian countries reported the lowest male mortality rates with ASMRs ranging from 1.44 to 2.46 per 100 000. In Europe, only Switzerland reported a rate below 2 per 100 000 males. Compared with the 5-year period 2010–2014, appreciable declines in ASMRs were observed in most countries considered. Exceptions include Bulgaria, Portugal, Romania, and several Latin American countries: Brazil, Chile, Colombia, and Mexico. Among females, similar to males, the highest ASMRs in 2020 were in the Czech Republic (2.15/100 000) and Hungary (2.10/100 000), while the remaining countries had rates below 2 per 100 000 females. The lowest rates were recorded in the Republic of Korea (0.43/100 000), Japan (0.48/100 000), and Hong Kong SAR (0.65/100 000). Again, most countries analysed showed favourable trends in mortality compared with the 2010–2014 period, while increases were reported in Bulgaria, Portugal, Brazil, Chile, and Colombia. Figure 1 is the corresponding bar plot ordered from the highest to the lowest male rate in 2020. Supplementary Table S1, Supplemental digital content 1, https://links.lww.com/EJCP/A581 reports the number of deaths and ASMRs in 2010–2014 and 2020 in the following age groups: 25–49, 50–69, and 70+ years. In most countries, recent trends have tended to be more favourable for young- and middle-age adults than for older people. Figure 2 shows the trends in ASMRs since 2000 for the 21 major countries worldwide and the EU-27. It also includes predicted rates for 2025 (for countries with ≥20 million inhabitants and the EU-27), with 95% prediction intervals for both males and females at all ages. Most trends were favourable except for Latin America, where, however, rates of Mexico and Brazil remained comparatively low.

**Table 1:** Age-standardised mortality rates from kidney cancer at all ages per 100 000 males and females in 2010–2014 and 2020 (unless indicated in parenthesis), annual average deaths, and the corresponding percent change in rates in selected countries worldwide

	Males					Females				
	Annual average deaths 2010–2014	ASMR 2010–2014	Deaths 2020	ASMR 2020	% Change	Annual average deaths 2010–2014	ASMR 2010–2014	Deaths 2020	ASMR 2020	% Change
Europe										
Austria	219	2.53	219	2.01	−20.6	191	1.50	156	0.90	−40.0
Belgium	342	2.96	307	2.32	−21.6	225	1.32	205	1.13	−14.4
Bulgaria	225	3.36	279	3.80	13.1	101	1.09	111	1.24	13.8
Czech Republic	639	6.68	582	5.13	−23.2	402	2.82	335	2.15	−23.8
Denmark	183	3.27	149	2.23	−31.8	103	1.43	93	1.02	−28.7
Finland	207	3.61	201	2.90	−19.7	156	1.81	152	1.54	−14.9
France	2292	3.52	2231	2.87	−18.5	1257	1.28	1203	1.01	−21.1
Germany	3209	3.38	3121	2.65	−21.6	2104	1.47	2034	1.18	−19.7
Hungary	455	5.53	463	5.03	−9.0	302	2.19	297	2.10	−4.1
Italy	2146	3.02	2352	2.76	−8.6	1199	1.19	1206	1.03	−13.4
Netherlands	593	3.58	607	2.87	−19.8	354	1.67	316	1.15	−31.1
Norway (2016)	177	3.77	158	2.88	−23.6	98	1.46	87	1.25	−14.4
Poland	1585	5.24	1434	3.93	−25.0	973	2.10	946	1.71	−18.6
Portugal (2019)	254	2.36	296	2.43	3.0	138	0.81	158	0.90	11.1
Romania (2019)	496	3.03	565	3.26	7.6	273	1.18	284	1.11	−5.9
Slovakia	244	6.19	276	5.65	−8.7	145	2.39	136	1.80	−24.7
Spain	1330	2.84	1386	2.50	−12.0	679	1.04	730	0.96	−7.7
Sweden	307	2.74	301	2.30	−16.1	219	1.47	177	1.06	−27.9
Switzerland	197	2.36	164	1.56	−33.9	108	0.87	102	0.82	−5.7
UK	2373	3.74	2495	3.25	−13.1	1465	1.78	1568	1.62	−9.0
EU-27	15 973	3.58	15 999	3.01	−15.9	9497	1.46	9243	1.21	−17.1
North America										
Canada	1045	3.22	1096	2.58	−19.9	587	1.38	615	1.20	−13.0
USA	8697	3.34	9418	2.91	−12.9	4753	1.40	4817	1.22	−12.9
Latin America										
Argentina	1192	4.86	1358	4.74	−2.5	577	1.77	591	1.65	−6.8
Brazil	1642	1.78	2250	1.90	6.7	990	0.86	1380	0.92	7.0
Chile	439	3.95	597	4.02	1.8	243	1.72	330	1.79	4.1
Colombia	267	1.34	381	1.39	3.7	174	0.73	293	0.89	21.9
Mexico	1203	2.42	1789	2.74	13.2	747	1.31	989	1.31	0.0
Australasia										
Australia	581	2.89	590	2.21	−23.5	337	1.28	308	0.97	−24.2
Hong Kong SAR	102	1.55	133	1.54	−0.6	56	0.68	76	0.65	−4.4
Israel	139	2.74	154	2.46	−10.2	87	1.20	68	0.80	−33.3
Japan	2956	1.69	3063	1.44	−14.8	1426	0.55	1568	0.48	−12.7
Republic of Korea	627	1.80	764	1.51	−16.1	258	0.53	312	0.43	−18.9

ASMR, age-standardised mortality rate.

Supplementary Tables S3a and b, Supplemental digital content 1, https://links.lww.com/EJCP/A581 provide detailed results from the joinpoint analyses. All countries considered showed a favourable trend over time for both males and females, except for Romania, which reported an increase of +2.0% per year in males and +0.9% in females, and Latin American countries. For males, the largest decreases were observed in Poland (AAPC: −4.1%), the Republic of Korea (−3.4%), the Czech Republic (−3.3%), and Sweden (−3.0%), while for females in Sweden (−4.1%), France (−3.7%), the Czech Republic (−3.4%), and Belgium (−3.0%). Table 2 provides the predicted number of deaths and the corresponding estimated ASMRs per 100 000 in 2025 from kidney cancer at all ages in selected major countries according to sex, along with comparison figures for 2020 and the percent change in rates. Among males, all considered countries are predicted to show a decline in ASMRs by 2025, except for Spain and Australia. Germany, Poland, and Japan are predicted to show the largest declines. The EU-27 is expected to experience a 14% decrease, from 3.01 to 2.60 per 100 000 males. Among females, similar downward mortality rates are expected in all countries, with Germany, Poland, and Japan showing marked reductions. The ASMR in the EU-27 is expected to reduce from 1.21 in 2020 to 1.04 per 100 000 females in 2025 (−14.0%). In terms of absolute numbers, the USA is projected to account for 9400 male and 4900 female deaths in 2025, and the EU-27 for 15 100 male and 8800 female deaths.

**Table 2:** Number of predicted deaths and age-standardised mortality rates from kidney cancer at all ages for the year 2025 and comparison figures for 2020, for selected countries worldwide and the European Union as a whole, with 95% prediction intervals

	Males					Females				
	Annual average deaths 2020	Predicted number of deaths 2025 (95% PI)	Observed ASMR 2020	Predicted ASMR 2025 (95% PI)	% Difference 2025 vs. 2020	Annual average deaths 2020	Predicted number of deaths 2025 (95% PI)	Observed ASMR 2020	Predicted ASMR 2025 (95% PI)	% Difference 2025 vs. 2020
France	2231	2100 (1900–2200)	2.87	2.49 (2.32–2.65)	−13.4	1203	1000 (900–1200)	1.01	0.82 (0.71–0.93)	−18.9
Germany	3121	2900 (2700–3200)	2.65	2.14 (1.92–2.36)	−19.3	2034	1800 (1600–1900)	1.18	0.90 (0.79–1.01)	−24
Italy	2352	2300 (2200–2500)	2.76	2.52 (2.34–2.70)	−8.7	1206	1300 (1200–1400)	1.03	0.98 (0.86–1.09)	−5.3
Poland	1434	1200 (1100–1300)	3.93	2.98 (2.69–3.27)	−24.2	946	800 (690–910)	1.71	1.26 (1.07–1.45)	−26.3
Spain	1386	1500 (1400–1600)	2.50	2.63 (2.46–2.81)	5.3	730	780 (720–840)	0.96	0.92 (0.81–1.04)	−3.7
UK	2495	2500 (2400–2700)	3.25	3.09 (2.88–3.30)	−5.1	1568	1600 (1500–1700)	1.62	1.55 (1.42–1.67)	−4.5
EU-27	15 999	15 100 (14 600–15 600)	3.01	2.60 (2.49–2.72)	−13.5	9243	8800 (8500–9100)	1.21	1.04 (0.98–1.10)	−14.0
Canada	1096	1100 (1000–1200)	2.58	2.25 (2.04–2.46)	−12.8	615	590 (530–660)	1.20	1.01 (0.89–1.14)	−15.5
USA	9418	9400 (9100–9700)	2.91	2.58 (2.49–2.67)	−11.2	4817	4900 (4700–5100)	1.22	1.12 (1.06–1.17)	−8.5
Argentina	1358	1400 (1300–1500)	4.74	4.57 (4.25–4.89)	−3.5	591	620 (550–690)	1.65	1.59 (1.41–1.77)	−3.6
Brazil	2250	2600 (2500–2700)	1.90	1.85 (1.76–1.95)	−2.5	1380	1600 (1500–1700)	0.92	0.90 (0.84–0.95)	−2.5
Colombia	381	440 (400–480)	1.39	1.38 (1.25–1.52)	−0.5	293	290 (260–320)	0.89	0.74 (0.66–0.83)	−16.5
Mexico	1789	1900 (1800–2000)	2.74	2.57 (2.43–2.71)	−6.3	989	1100 (1000–1200)	1.31	1.25 (1.17–1.33)	−4.3
Australia	590	690 (630–740)	2.21	2.26 (2.04–2.48)	2.2	308	310 (270–350)	0.97	0.86 (0.73–0.98)	−11.8
Japan	3063	2900 (2800–3100)	1.44	1.17 (1.08–1.27)	−18.6	1568	1400 (1300–1500)	0.48	0.34 (0.28–0.40)	−29.2
Republic of Korea	764	840 (760–910)	1.51	1.37 (1.24–1.51)	−9.0	312	360 (310–400)	0.43	0.42 (0.34–0.50)	−2.3

ASMR, age-standardised mortality rate; PI, prediction interval.

Table 3 gives the ASIRs from kidney cancer per 100 000 males and females across selected countries, comparing two time periods: 2005–2007 and 2015–2017. Among males, incidence rates increased in all countries (except for Poland), with notable rises observed in Japan (+54.9% vs 2005–2007), Denmark (+40.3%), the UK (+38.4%), and Norway (+35.4%). Modest increases were observed in Germany (+4.5%) and Switzerland (+4.8%). Similar trends were observed among females, although incidence rates were lower. Large relative increases in ASIRs were recorded in Japan (+44.8% vs. 2005–2007), Denmark (+37.4%), and the UK (+39.8%). Other notable increases occurred in Norway (+17.4%), Australia (+20.5%), and Italy (+11.0%). Supplementary Table S2, Supplemental digital content 1, https://links.lww.com/EJCP/A581 reports corresponding figures across different age groups: 25–49, 50–69, and 70+ years.

**Table 3:** Age-standardised incidence rates in selected countries from kidney cancer per 100 000 males and females in 2005–2007and 2015–2017^a^, annual average deaths at all ages and the corresponding percent change in rates.

	Males					Females				
	Annual average incidence cases 2005–2007	ASIR 2005–2007	Annual average incidence cases 2015–2017	ASIR 2015–2017	% Change	Annual average incidence cases 2005–2007	ASIR 2005–2007	Annual average incidence cases 2015–2017	ASIR 2015–2017	% Change
Denmark	390	8.76	478	12.29	40.3	217	4.14	245	5.69	37.4
France	762	12.21	549	14.68	20.2	412	5.45	264	5.79	6.2
Germany	577	10.22	521	10.68	4.5	347	4.80	277	4.90	2.1
Italy	275	9.49	195	11.47	20.9	163	4.45	105	4.94	11.0
Netherlands	1185	9.29	1255	10.36	11.5	760	5.14	708	5.46	6.2
Norway	376	10.04	453	13.59	35.4	236	5.52	228	6.48	17.4
Poland	95	10.83	83	10.57	-2.4	57	4.94	61	6.43	30.2
Spain	588	8.94	407	10.56	18.1	291	3.77	176	4.18	10.9
Switzerland	145	9.38	143	9.83	4.8	77	3.93	62	3.84	−2.3
UK	4395	8.74	5300	12.10	38.4	2666	4.55	3158	6.36	39.8
Canada	1808	10.42	2187	12.96	24.4	1128	5.74	1163	6.41	11.7
USA	2387	12.94	2443	14.06	8.7	1470	6.78	1335	7.01	3.4
Argentina	72	8.24	88	11.77	42.8	45	4.48	43	4.79	6.9
Chile	22	10.51	25	11.26	7.1	12	5.51	13	5.33	−3.3
Colombia	61	3.81	92	5.53	45.1	45	2.17	69	3.30	52.1
Australia	1663	11.03	1812	12.85	16.5	858	5.16	933	6.22	20.5
Japan	642	6.12	274	9.48	54.9	306	2.32	120	3.36	44.8
Republic of Korea	1817	6.53	2583	8.74	33.8	838	2.66	1205	3.71	39.5

National figures were estimated from regional registries: France: Bas-Rhin, Calvados, Doubs, Haut-Rhin, Isère, Somme, Hérault, Loire-Atlantique, Manche, Vendée; Germany: Hamburg, Bremen, Schleswig-Holstein, and Saarland; Italy: Umbria (Perugia), Trento, Syracuse, Palermo, and South Tyrol; Poland: Kielce; Spain: Tarragona, Granada, Murcia, Navarra, Basque Country, Girona, Canary Islands, and La Rioja; Switzerland: Geneva, Vaud, Valais, Ticino, Graubünden and Glarus; UK: England, Scotland, Wales, Northern Ireland; Chile: Valdivia; Canada: Nova Scotia, Quebec, Northwest Territories, Nunavut, and Yukon; Argentina: Mendoza; Colombia: Cali, Bucaramanga, Manizales, Pasto; Australia: New South Wales & the Australian Capital Territory, Queensland, South Australia, Tasmania, Victoria, Western Australia, and the Northern Territor; Japan: Miyagi Prefecture and Osaka.

ASIR, age-standardised incidence rate.

a Data for France, Italy, and Spain are available up to 2016, while data for Japan are available up to 2015.

**Fig. 3:**
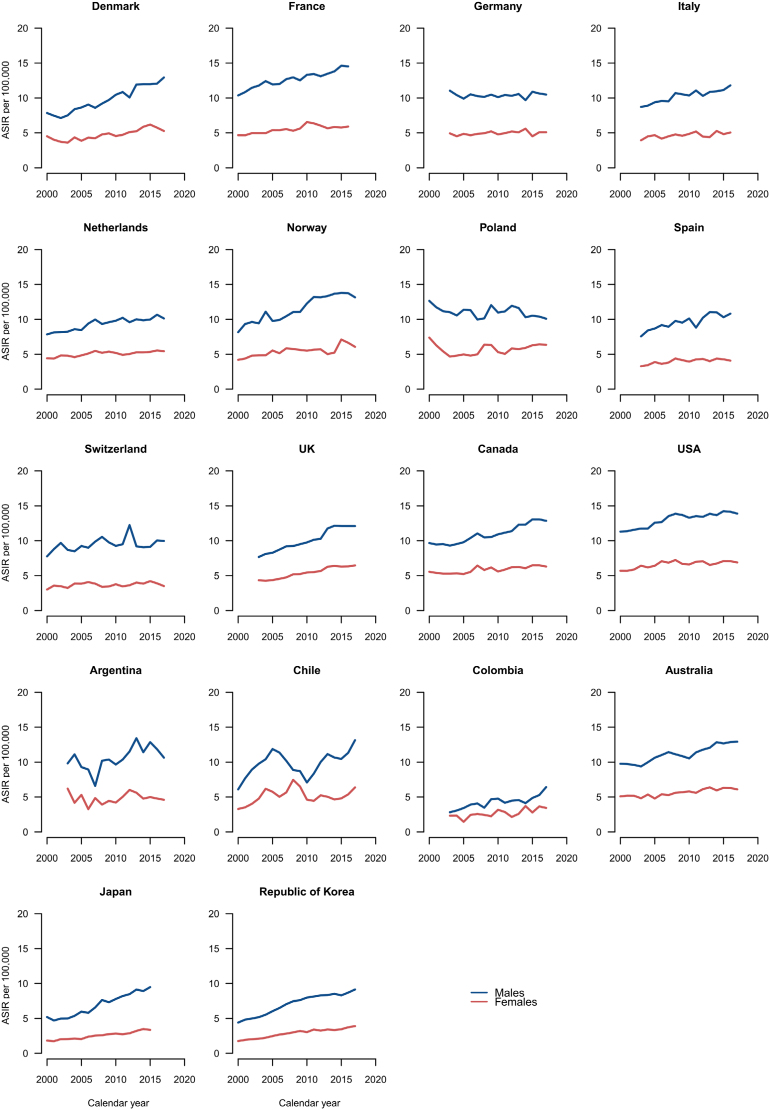
Annual ASIR per 100 000 males and females at all ages from kidney cancer in major countries worldwide. ASIR, age-standardised incidence rate.

Trends in ASIRs per 100 000 males and females from 1990 to 2017 estimated using LOESS regression, are shown in Fig. 3. Incidence rates increased over the entire period in both sexes across all countries considered, except in Germany and Switzerland.

## Discussion

Our findings show that kidney cancer mortality rates generally decreased worldwide, with the main exceptions of Latin America and Eastern Europe, which also had the highest mortality rates. However, due to population growth or ageing, the absolute number of deaths from kidney cancer remained stable or even increased slightly in several countries. On the other hand, kidney cancer incidence increased for both sexes in most high-income countries over the study period.

Renal cell carcinoma (RCC) currently accounts for more than 90% of kidney cancers (Hsieh *et al*., 2017; Bahadoram *et al*., 2022). RCC has a genetic and metabolic basis: it can arise from germline or somatic mutations that can alter metabolic pathways (Linehan *et al*., 2019; Larcher *et al*., 2025). Mutations in genes associated with the development of kidney cancer can affect the cell’s ability to respond to changes in oxygen levels, iron availability, and nutrients (Schmidt and Linehan, 2016). Identified risk factors for kidney cancer include overweight, history of hypertension and smoking, which are estimated to account for up to 50% of cases of RCC overall (Benichou *et al*., 1998). In particular, a population-based case-control study of RCC conducted in Minnesota reported attributable risks of 21% for hypertension, 21% for overweight, and 18% for smoking, including both past and current smokers (Benichou *et al*., 1998). Two subsequent studies in Italy estimated that 17% of kidney cancer cases were due to overweight and obesity (Di Maso *et al*., 2023), and 12% to smoking (Collatuzzo *et al*., 2024). Other risk factors are chronic kidney disease and end-stage renal disease (Bukavina *et al*., 2022).

A study on the incidence and mortality of RCC in the US revealed that, from 2008 to 2019, the incidence rate exhibited a gradual increase of 1.1% per year; however, the incidence-based mortality tended to decrease (Chen *et al*., 2025). Nationwide registry data from Denmark covering all patients diagnosed with primary RCC from 1992 to 2021, indicated that the incidence of RCC has increased over the past three decades, with the proportion of male patients rising; this increase was driven by a growing number of localised cases, while the proportion of metastatic cases has decreased and survival for metastatic RCC has improved (Ahrenfeldt *et al*., 2025). A study based on incidence and mortality data from the Cancer Today tool and survival estimates from international, national, or subnational population-based cancer registries revealed that 434 840 new cases of kidney cancer and 155 953 associated deaths were recorded worldwide in 2022 (Larcher *et al*., 2025). Notably, there was significant geographic variability in both incidence and mortality rates. Five-year overall survival rates ranged from 40 to 63% in Africa, 66–76% in Europe, 67% in North America, 69% in Asia, 75% in Oceania, and up to 86% in nonmetastatic cases in Latin America (Larcher *et al*., 2025).

The increasing incidence of kidney cancer in Western countries can also be related to the widespread diffusion of diagnostic procedures, mainly abdominal ultrasound, computed tomography, and MRI. These imaging modalities are frequently performed for unrelated abdominal symptoms or as part of routine health check-ups, leading to the incidental detection of small renal masses, many of which are asymptomatic and clinically indolent. This leads to a stage migration phenomenon, which resulted in a shift toward the diagnosis of early-stage, localised tumours that would have otherwise remained undetected. Therefore, a proportion of the increase in kidney cancer incidence observed in high-income countries is attributable to greater imaging utilisation rather than a true rise in disease burden.

High rates and unfavourable mortality trends were observed in Eastern Europe, in particular in Romania, and in Latin America. These could reflect a high prevalence of smoking over recent decades in Central and Eastern Europe (EUROSTAT, 2022), a high prevalence of overweight and obesity, as well as, and inadequate control of hypertension in Latin America (Padala *et al*., 2020; Champagne *et al*., 2022; Ferreira *et al*., 2024). In addition, the unfavourable trend in Romania could reflect the presence of Balkan endemic nephropathy (BEN) (Lukinich-Gruia *et al*., 2022), associated with aristolochic acid. Nuclear adducts of aristolochic acid were found in renal tissues and upper tract urothelial carcinoma among patients with BEN. Aristolochic acid was classified as a group 1 carcinogenic and toxic compound by the International Agency for Research on Cancer in 2002 (IARC Working Group on the Evaluation of Carcinogenic Risks to Humans, 2002). Aristolochic acid was first identified as a cause of kidney failure in the early 1990s, following the use of aristolochic acid–contaminated diet pills in Belgium and other Western European countries (Vanherweghem *et al*., 1993). Traditionally, *Aristolochia*-based remedies have been used in herbal medicine in Romania. Additional environmental exposure in the Balkans has been related to *Aristolochia clematitis*, a plant that grows widely in agricultural fields and along rivers, thus enabling aristolochic acid to infiltrate the food chain through contaminated crops, soil, and water (Tung *et al*., 2020; Draghia *et al*., 2021; Guo *et al*., 2021).

We observed a general decrease in mortality in high-income countries. These trends are likely attributable to the control of tobacco and hypertension. In addition, the availability and widespread use of abdominal imaging, which is often performed during opportunistic check-ups or for unrelated abdominal conditions, resulted in a stage migration phenomenon, with the identification of small renal masses that can be surgically removed or observed without posing the patient at risk of dying from kidney cancer. In addition, the therapeutic landscape of advanced kidney cancer has undergone substantial transformation over the past two decades, particularly with the advent of targeted therapies – including tyrosine kinase inhibitors – and more recently, immune checkpoint inhibitors (Ljungberg *et al*., 2022; Allaf *et al*., 2024). These systemic agents have been shown to significantly prolong progression-free and overall survival in patients with metastatic RCC, a population that previously had limited treatment options and poor prognosis (Ljungberg *et al*., 2022). As such, improvements in systemic therapy, coupled with a multidisciplinary approach, likely contributed to the decline in mortality, even among patients diagnosed at more advanced stages who are not candidates for local therapies.

Incidence and mortality rates were about twice as high in males as in females. Males have a higher risk of developing kidney cancer and of being diagnosed with a more aggressive form of the disease, and their prognosis is generally worse than that of females (Lughezzani *et al*., 2019). Differences in risk factors, exposure, genetics, and sex hormones may contribute to this pattern, although the biological mechanisms underlying these differences are not fully understood (Peired *et al*., 2021).

Mortality predictions should be interpreted with caution, as they assume that current trends in risk factors, screening effectiveness, and treatment practices will remain unchanged over time. Any change in these parameters could significantly affect future outcomes, although this are less of a problem for short-term projections. Additional potential limitations include the validity of death certification, coding errors, and misclassification of cancer types, particularly when comparing data across countries that have transitioned between different versions of the International Classification of Diseases in different periods.

### Conclusion

We found different trends between kidney cancer incidence and mortality. Kidney cancer mortality declined globally, except for Latin America and a few countries in Eastern Europe. These patterns likely reflect improvements in early diagnosis and treatment, smoking cessation mainly among men, and better control of hypertension. Incidence of kidney cancer has been rising in most high-income countries over the study period, likely reflecting widespread increases in the use of imaging techniques.

## Acknowledgements

This work was supported by the AIRC Foundation, Italy (Grant no. 22987 to E.N.). C.S. and C.L.V. were also supported by EU funding within the NextGenerationEU-MUR PNRR Extended Partnership initiative (no. PE00000007, INF-ACT).

### Conflicts of interest

There are no conflicts of interest.

## Supplementary Material
