# Supplementary material for: The global burden of kidney cancer: trends in mortality and incidence with predictions to 2025
Source: Eur J Cancer Prev. 2025 Dec 16;35(5):395–404. doi: 10.1097/CEJ.0000000000001000 (PMC13412325; doi:10.1097/CEJ.0000000000001000)
Supplement: Supplementary file 1 [file ejcp-35-395-s001.docx]

**SUPPLEMENTARY MATERIAL**

**Manuscript title:**

The Global Burden of Kidney Cancer: Trends in Mortality and Incidence with Predictions to 2025

**Table of contents**

[**Table S1.** Age-standardized mortality rates (ASMR) from kidney cancer per 100,000 males and females in 2010-14 and 2020, annual average deaths and the corresponding percent change in rates in selected countries worldwide according to different age groups. 2](#_Toc198036029)

[**Table S2.** Age-standardized incidence rates (ASIR) from kidney cancer per 100,000 males and females in 2005-07 and 2015-17, annual average deaths and the corresponding percent change in rates in selected countries worldwide according to different age groups. 3](#_Toc198036030)

[**Table S3a.** Joinpoint analysis results for kidney cancer since 2000 (2001 for the UK) to the latest available year in selected countries worldwide, for all among males. 4](#_Toc198036031)

[**Table S3b.** Joinpoint analysis results for kidney cancer since 2000 (2001 for the UK) to the latest available year in selected countries worldwide, for all among females. 5](#_Toc198036032)

# **Table S1.** Age-standardized mortality rates (ASMR) from kidney cancer per 100,000 males and females in 2010-14 and 2020, annual average deaths and the corresponding percent change in rates in selected countries worldwide according to different age groups.

| **Country**  **Age group** | **Males** | | | | |  | **Females** | | | | |
| --- | --- | --- | --- | --- | --- | --- | --- | --- | --- | --- | --- |
|  | **Annual average deaths**  **2010-14** | **ASMR 2010-14** | **Deaths 2020** | **ASMR 2020** | **% change** |  | **Annual average deaths**  **2010-14** | **ASMR 2010-14** | **Deaths 2020** | **ASMR 2020** | **% change** |
| France |  |  |  |  |  |  |  |  |  |  |  |
| *25-49* | 94 | 0.85 | 78 | 0.74 | -12.9 |  | 40 | 0.35 | 21 | 0.19 | -45.7 |
| *50-69* | 792 | 10.45 | 682 | 7.99 | -23.5 |  | 279 | 3.42 | 227 | 2.43 | -28.9 |
| *70+* | 1399 | 38.39 | 1465 | 31.99 | -16.7 |  | 934 | 14.80 | 949 | 12.85 | -13.2 |
| Germany |  |  |  |  |  |  |  |  |  |  |  |
| *25-49* | 104 | 0.60 | 45 | 0.33 | -45.0 |  | 39 | 0.24 | 19 | 0.14 | -41.7 |
| *50-69* | 1021 | 9.87 | 907 | 7.50 | -24.0 |  | 408 | 3.77 | 373 | 2.94 | -22.0 |
| *70+* | 2080 | 39.25 | 2165 | 33.21 | -15.4 |  | 1653 | 19.28 | 1638 | 16.31 | -15.4 |
| Italy |  |  |  |  |  |  |  |  |  |  |  |
| *25-49* | 91 | 0.74 | 83 | 0.72 | -2.7 |  | 36 | 0.29 | 34 | 0.30 | 3.4 |
| *50-69* | 665 | 8.87 | 623 | 7.53 | -15.1 |  | 259 | 3.21 | 218 | 2.45 | -23.7 |
| *70+* | 1384 | 33.37 | 1644 | 32.79 | -1.7 |  | 898 | 13.50 | 949 | 12.78 | -5.3 |
| Poland |  |  |  |  |  |  |  |  |  |  |  |
| *25-49* | 80 | 1.20 | 60 | 0.78 | -35.0 |  | 25 | 0.38 | 17 | 0.23 | -39.5 |
| *50-69* | 796 | 17.79 | 642 | 12.37 | -30.5 |  | 332 | 6.47 | 282 | 4.75 | -26.6 |
| *70+* | 708 | 49.81 | 729 | 41.62 | -16.4 |  | 613 | 22.91 | 643 | 20.86 | -8.9 |
| Spain |  |  |  |  |  |  |  |  |  |  |  |
| *25-49* | 79 | 0.79 | 75 | 0.76 | -3.8 |  | 28 | 0.30 | 28 | 0.29 | -3.3 |
| *50-69* | 458 | 8.78 | 449 | 7.30 | -16.9 |  | 167 | 2.99 | 160 | 2.47 | -17.4 |
| *70+* | 789 | 28.96 | 858 | 26.19 | -9.6 |  | 481 | 10.89 | 538 | 10.93 | 0.4 |
| UK |  |  |  |  |  |  |  |  |  |  |  |
| *25-49* | 117 | 0.98 | 85 | 0.75 | -23.5 |  | 49 | 0.41 | 40 | 0.35 | -14.6 |
| *50-69* | 856 | 11.36 | 766 | 9.43 | -17.0 |  | 391 | 4.96 | 366 | 4.30 | -13.3 |
| *70+* | 1394 | 39.75 | 1640 | 37.06 | -6.8 |  | 1018 | 20.37 | 1154 | 19.51 | -4.2 |
| EU-27 |  |  |  |  |  |  |  |  |  |  |  |
| *25-49* | 721 | 0.84 | 534 | 0.65 | -22.6 |  | 263 | 0.31 | 199 | 0.24 | -22.6 |
| *50-69* | 5986 | 11.19 | 5381 | 9.01 | -19.5 |  | 2368 | 4.07 | 2030 | 3.14 | -22.9 |
| *70+* | 9230 | 37.31 | 10052 | 33.42 | -10.4 |  | 6830 | 16.81 | 6975 | 14.95 | -11.1 |
| Canada |  |  |  |  |  |  |  |  |  |  |  |
| *25-49* | 49 | 0.72 | 33 | 0.52 | -27.8 |  | 16 | 0.25 | 14 | 0.22 | -12.0 |
| *50-69* | 424 | 9.79 | 380 | 7.36 | -24.8 |  | 167 | 3.74 | 177 | 3.31 | -11.5 |
| *70+* | 571 | 35.30 | 680 | 29.98 | -15.1 |  | 402 | 17.04 | 421 | 14.37 | -15.7 |
| USA |  |  |  |  |  |  |  |  |  |  |  |
| *25-49* | 492 | 0.87 | 404 | 0.72 | -17.2 |  | 188 | 0.33 | 177 | 0.32 | -3.0 |
| *50-69* | 3844 | 10.84 | 3680 | 8.90 | -17.9 |  | 1579 | 4.12 | 1489 | 3.39 | -17.7 |
| *70+* | 4326 | 32.47 | 5304 | 31.72 | -2.3 |  | 2956 | 15.33 | 3117 | 13.90 | -9.3 |
| Argentina |  |  |  |  |  |  |  |  |  |  |  |
| *25-49* | 81 | 1.27 | 80 | 1.04 | -18.1 |  | 38 | 0.59 | 45 | 0.58 | -1.7 |
| *50-69* | 587 | 17.51 | 621 | 16.55 | -5.5 |  | 227 | 5.92 | 238 | 5.55 | -6.3 |
| *70+* | 514 | 42.60 | 645 | 45.60 | 7.0 |  | 304 | 14.84 | 301 | 13.01 | -12.3 |
| Brazil |  |  |  |  |  |  |  |  |  |  |  |
| *25-49* | 173 | 0.49 | 175 | 0.45 | -8.2 |  | 108 | 0.29 | 109 | 0.27 | -6.9 |
| *50-69* | 813 | 5.61 | 1096 | 5.80 | 3.4 |  | 409 | 2.49 | 573 | 2.66 | 6.8 |
| *70+* | 614 | 17.00 | 947 | 19.71 | 15.9 |  | 429 | 8.00 | 659 | 8.98 | 12.3 |
| Colombia |  |  |  |  |  |  |  |  |  |  |  |
| *25-49* | 28 | 0.35 | 29 | 0.34 | -2.9 |  | 19 | 0.23 | 25 | 0.28 | 21.7 |
| *50-69* | 135 | 4.30 | 193 | 4.59 | 6.7 |  | 73 | 2.02 | 121 | 2.45 | 21.3 |
| *70+* | 93 | 12.16 | 156 | 14.16 | 16.4 |  | 72 | 6.73 | 136 | 8.75 | 30.0 |
| Mexico |  |  |  |  |  |  |  |  |  |  |  |
| *25-49* | 152 | 0.86 | 208 | 1.00 | 16.3 |  | 83 | 0.42 | 96 | 0.42 | 0.0 |
| *50-69* | 589 | 8.20 | 857 | 9.03 | 10.1 |  | 340 | 4.26 | 472 | 4.33 | 1.6 |
| *70+* | 439 | 19.14 | 686 | 22.81 | 19.2 |  | 297 | 10.74 | 399 | 10.88 | 1.3 |
| Australia |  |  |  |  |  |  |  |  |  |  |  |
| *25-49* | 32 | 0.79 | 17 | 0.38 | -51.9 |  | 13 | 0.32 | 7 | 0.15 | -53.1 |
| *50-69* | 231 | 8.97 | 185 | 6.26 | -30.2 |  | 81 | 3.08 | 81 | 2.56 | -16.9 |
| *70+* | 317 | 29.44 | 388 | 26.85 | -8.8 |  | 241 | 16.27 | 217 | 11.67 | -28.3 |
| Japan |  |  |  |  |  |  |  |  |  |  |  |
| *25-49* | 93 | 0.41 | 69 | 0.30 | -26.8 |  | 36 | 0.17 | 35 | 0.15 | -11.8 |
| *50-69* | 946 | 5.15 | 710 | 4.13 | -19.8 |  | 269 | 1.41 | 200 | 1.12 | -20.6 |
| *70+* | 1913 | 18.20 | 2276 | 16.41 | -9.8 |  | 1116 | 6.34 | 1329 | 5.83 | -8.0 |
| Republic of Korea |  |  |  |  |  |  |  |  |  |  |  |
| *25-49* | 50 | 0.45 | 47 | 0.42 | -6.7 |  | 18 | 0.17 | 12 | 0.12 | -29.4 |
| *50-69* | 272 | 5.04 | 289 | 3.92 | -22.2 |  | 66 | 1.18 | 79 | 1.03 | -12.7 |
| *70+* | 303 | 20.42 | 427 | 18.67 | -8.6 |  | 171 | 6.52 | 221 | 5.62 | -13.8 |

# **Table S2.** Age-standardized incidence rates (ASIR) from kidney cancer per 100,000 males and females in 2005-07 and 2015-17^a^, annual average deaths and the corresponding percent change in rates in selected countries worldwide according to different age groups.

| **Country**  **Age group** | **Males** | | | | |  | **Females** | | | | |
| --- | --- | --- | --- | --- | --- | --- | --- | --- | --- | --- | --- |
|  | **Annual average incidence cases**  **2005-07** | **ASIR**  **2005-07** | **Annual average incidence cases**  **2015-17** | **ASIR**  **2015-17** | **% change** |  | **Annual average incidence cases**  **2005-07** | **ASIR**  **2005-07** | **Annual average incidence cases**  **2015-17** | **ASIR**  **2015-17** | **% change** |
| France |  |  |  |  |  |  |  |  |  |  |  |
| *25-49* | 92 | 6.26 | 61 | 8.20 | 31.0 |  | 42 | 2.86 | 26 | 3.50 | 22.4 |
| *50-69* | 371 | 43.24 | 277 | 50.72 | 17.3 |  | 161 | 17.49 | 109 | 18.86 | 7.8 |
| *70+* | 292 | 75.73 | 416 | 91.80 | 21.2 |  | 201 | 34.09 | 255 | 37.30 | 9.4 |
| Germany |  |  |  |  |  |  |  |  |  |  |  |
| *25-49* | 64 | 4.85 | 52 | 5.76 | 18.8 |  | 27 | 2.08 | 22 | 2.50 | 20.2 |
| *50-69* | 287 | 35.04 | 225 | 35.81 | 2.2 |  | 129 | 15.34 | 98 | 14.88 | -3.0 |
| *70+* | 222 | 68.89 | 323 | 74.24 | 7.8 |  | 188 | 35.37 | 204 | 33.71 | -4.7 |
| Italy |  |  |  |  |  |  |  |  |  |  |  |
| *25-49* | 36 | 5.40 | 22 | 6.20 | 14.8 |  | 17 | 2.48 | 12 | 3.25 | 31.0 |
| *50-69* | 135 | 33.70 | 91 | 39.14 | 16.1 |  | 61 | 13.91 | 40 | 16.29 | 17.1 |
| *70+* | 103 | 53.83 | 162 | 69.29 | 28.7 |  | 83 | 29.59 | 106 | 30.89 | 4.4 |
| Poland |  |  |  |  |  |  |  |  |  |  |  |
| *25-49* | 10 | 4.20 | 7 | 4.28 | 1.9 |  | 6 | 2.42 | 4 | -^b^ | - |
| *50-69* | 53 | 40.51 | 49 | 37.72 | -6.9 |  | 24 | 16.08 | 33 | 23.17 | 44.1 |
| *70+* | 30 | 62.63 | 35 | 66.68 | 6.5 |  | 26 | 29.92 | 32 | 38.05 | 27.2 |
| Spain |  |  |  |  |  |  |  |  |  |  |  |
| *25-49* | 73 | 4.09 | 54 | 5.64 | 37.9 |  | 36 | 2.12 | 25 | 2.76 | 30.2 |
| *50-69* | 254 | 29.99 | 192 | 36.30 | 21.0 |  | 100 | 11.28 | 71 | 12.88 | 14.2 |
| *70+* | 253 | 60.81 | 314 | 65.08 | 7.0 |  | 150 | 24.54 | 155 | 22.86 | -6.8 |
| UK |  |  |  |  |  |  |  |  |  |  |  |
| *25-49* | 415 | 3.62 | 557 | 6.42 | 77.3 |  | 229 | 1.99 | 287 | 3.27 | 64.3 |
| *50-69* | 1931 | 28.57 | 2373 | 39.91 | 39.7 |  | 1016 | 14.40 | 1234 | 19.84 | 37.8 |
| *70+* | 2002 | 67.90 | 3099 | 83.19 | 22.5 |  | 1369 | 32.14 | 2111 | 43.45 | 35.2 |
| Canada |  |  |  |  |  |  |  |  |  |  |  |
| *25-49* | 260 | 5.19 | 265 | 7.46 | 43.7 |  | 142 | 2.88 | 142 | 3.91 | 35.8 |
| *50-69* | 913 | 36.73 | 1158 | 44.18 | 20.3 |  | 489 | 18.86 | 564 | 20.82 | 10.4 |
| *70+* | 616 | 64.48 | 996 | 79.74 | 23.7 |  | 475 | 36.54 | 587 | 37.56 | 2.8 |
| USA |  |  |  |  |  |  |  |  |  |  |  |
| *25-49* | 346 | 6.29 | 332 | 8.36 | 32.9 |  | 221 | 4.00 | 184 | 4.61 | 15.3 |
| *50-69* | 1241 | 46.25 | 1318 | 48.37 | 4.6 |  | 629 | 21.61 | 654 | 22.36 | 3.5 |
| *70+* | 774 | 80.77 | 1031 | 83.86 | 3.8 |  | 589 | 42.37 | 624 | 38.22 | -9.8 |
| Argentina |  |  |  |  |  |  |  |  |  |  |  |
| *25-49* | 9 | 3.70 | 13 | 6.73 | 81.9 |  | 5 | 1.98 | 6 | 2.55 | 28.8 |
| *50-69* | 36 | 28.81 | 46 | 40.45 | 40.4 |  | 23 | 15.68 | 22 | 16.49 | 5.2 |
| *70+* | 26 | 55.68 | 36 | 72.94 | 31.0 |  | 15 | 23.97 | 20 | 26.41 | 10.2 |
| Colombia |  |  |  |  |  |  |  |  |  |  |  |
| *25-49* | 15 | 2.42 | 15 | 2.74 | 13.2 |  | 8 | 1.11 | 11 | 1.81 | 63.1 |
| *50-69* | 29 | 12.42 | 52 | 19.54 | 57.3 |  | 20 | 6.59 | 33 | 9.45 | 43.4 |
| *70+* | 12 | 17.70 | 27 | 26.27 | 48.4 |  | 14 | 13.45 | 27 | 17.97 | 33.6 |
| Australia |  |  |  |  |  |  |  |  |  |  |  |
| *25-49* | 234 | 5.99 | 233 | 7.30 | 21.9 |  | 113 | 2.84 | 126 | 3.85 | 35.6 |
| *50-69* | 791 | 36.77 | 922 | 44.63 | 21.4 |  | 342 | 15.77 | 430 | 20.08 | 27.3 |
| *70+* | 619 | 73.64 | 850 | 75.45 | 2.5 |  | 382 | 34.17 | 477 | 34.98 | 2.4 |
| Japan |  |  |  |  |  |  |  |  |  |  |  |
| *25-49* | 70 | 3.74 | 29 | 5.51 | 47.3 |  | 19 | 1.00 | 11 | 1.93 | 93.0 |
| *50-69* | 312 | 19.66 | 132 | 34.47 | 75.3 |  | 128 | 7.64 | 45 | 11.08 | 45.0 |
| *70+* | 258 | 42.08 | 451 | 51.50 | 22.4 |  | 156 | 16.90 | 254 | 20.21 | 19.6 |
| Republic of Korea |  |  |  |  |  |  |  |  |  |  |  |
| *25-49* | 478 | 4.21 | 587 | 7.04 | 67.2 |  | 186 | 1.71 | 219 | 2.76 | 61.4 |
| *50-69* | 950 | 21.93 | 1377 | 28.24 | 28.8 |  | 390 | 8.34 | 589 | 11.83 | 41.8 |
| *70+* | 363 | 36.97 | 797 | 43.67 | 18.1 |  | 235 | 13.58 | 503 | 17.83 | 31.3 |

^a^ Data for France, Italy, and Spain are available up to 2016, while data for Japan are available up to 2015.

^b^ Incidence rate was not calculated since there were less than 5 deaths.

National figures were estimated from regional registries (**France**: Bas-Rhin, Calvados, Doubs, Haut-Rhin, Isère, Somme, Hérault, Loire-Atlantique, Manche, Vendée; **Germany**: Hamburg, Bremen, Schleswig-Holstein, and Saarland; **Italy**: Umbria (Perugia), Trento, Syracuse, Palermo, and South Tyrol; **Poland**: Kielce; **Spain**: Tarragona, Granada, Murcia, Navarra, Basque Country, Girona, Canary Islands, and La Rioja; **Switzerland**: Geneva, Vaud, Valais, Ticino, Graubünden and Glarus; **United Kingdom**: England, Scotland, Wales, Northern Ireland; **Chile:** Valdivia; **Canada:** Nova Scotia, Quebec, Northwest Territories, Nunavut, and Yukon; **Argentina**: Mendoza; **Colombia**: Cali, Bucaramanga, Manizales, Pasto; **Australia**: New South Wales & the Australian Capital Territory, Queensland, South Australia, Tasmania, Victoria, Western Australia, and the Northern Territor; **Japan**:Miyagi Prefecture and Osaka.)

# **Table S3a.** Joinpoint analysis results for kidney cancer since 2000 (2001 for the UK) to the latest available year in selected countries worldwide, for all among males.

| **Country** | **Year 1** | **APC 1** | **Year 2** | **APC 2** | **Year 3** | **APC 3** | **AAPC** |
| --- | --- | --- | --- | --- | --- | --- | --- |
| Belgium | 2000-2021 | -2.18 * |  |  |  |  | -2,18 |
| Czech Republic | 2000-2023 | -3.34 * |  |  |  |  | -3,34 |
| France | 2000-2015 | -0.80 * | 2015-2022 | -3.34 * |  |  | -2,78 |
| Germany | 2000-2020 | -2.22 * |  |  |  |  | -2,22 |
| Italy | 2000-2021 | -1.02 * |  |  |  |  | -1,02 |
| Netherlands | 2000-2023 | -2.06 * |  |  |  |  | -2,06 |
| Poland | 2000-2016 | -1.29 * | 2016-2022 | -5.48 * |  |  | -4,11 |
| Portugal | 2000-2022 | 0.53 * |  |  |  |  | 0,53 |
| Romania | 2000-2019 | 2.01 * |  |  |  |  | 2,01 |
| Spain | 2000-2022 | -0.24 |  |  |  |  | -0,24 |
| Sweden | 2000-2023 | -3.04 * |  |  |  |  | -3,04 |
| UK | 2001-2021 | -0.70 * |  |  |  |  | -0,70 |
| EU | 2000-2007 | -1.47 * | 2007-2015 | -0.73 * | 2015-2020 | -3.08 * | -2,04 |
| Canada | 2000-2015 | -0.95 * | 2015-2022 | -3.3 * |  |  | -2,78 |
| USA | 2000-2015 | -0.93 * | 2015-2018 | -3.46 | 2018-2022 | -1.04 | -1,83 |
| Argentina | 2000-2022 | 0.37 * |  |  |  |  | 0,37 |
| Brazil | 2000-2004 | -15.65 * | 2004-2021 | 1.44 * |  |  | 1,44 |
| Chile | 2000-2021 | 0.68 * |  |  |  |  | 0,68 |
| Mexico | 2000-2022 | 1.05 * |  |  |  |  | 1,05 |
| Australia | 2000-2023 | -1.73 * |  |  |  |  | -1,73 |
| Japan | 2000-2015 | -0.48 * | 2015-2021 | -3.19 * |  |  | -2,30 |
| Republic of Korea | 2000-2013 | 0.40 | 2013-2022 | -3.41 * |  |  | -3,41 |

APC, annual percent change; AAPC, average annual percent change.

# **Table S3b.** Joinpoint analysis results for kidney cancer since 2000 (2001 for the UK) to the latest available year in selected countries worldwide, for all among females.

| **Country** | **Year 1** | **APC 1** | **Year 2** | **APC 2** | **Year 3** | **APC 3** | **Year 4** | **APC 4** | **Year 5** | **APC 5** | **AAPC** |
| --- | --- | --- | --- | --- | --- | --- | --- | --- | --- | --- | --- |
| Belgium | 2000-2021 | -3.03 * |  |  |  |  |  |  |  |  | -3,03 |
| Czech Republic | 2000-2023 | -3.40 * |  |  |  |  |  |  |  |  | -3,40 |
| France | 2000-2002 | 1.35 | 2002-2008 | -4.95 * | 2008-2011 | 3.86 | 2011-2017 | -1,23 | 2017-2022 | -5.57 * | -3,66 |
| Germany | 2000-2020 | -2.47 * |  |  |  |  |  |  |  |  | -2,47 |
| Italy | 2000-2021 | -0.83 * |  |  |  |  |  |  |  |  | -0,83 |
| Netherlands | 2000-2023 | -2.97 * |  |  |  |  |  |  |  |  | -2,97 |
| Poland | 2000-2012 | -1.25 * | 2012-2022 | -3.23 * |  |  |  |  |  |  | -3,23 |
| Portugal | 2000-2022 | 0.21 |  |  |  |  |  |  |  |  | 0,21 |
| Romania | 2000-2019 | 0.94 * |  |  |  |  |  |  |  |  | 0,94 |
| Spain | 2000-2022 | -0.63 * |  |  |  |  |  |  |  |  | -0,63 |
| Sweden | 2000-2023 | -4.10 * |  |  |  |  |  |  |  |  | -4,10 |
| UK | 2001-2004 | 3.56 | 2004-2021 | -1.16 * |  |  |  |  |  |  | -1,16 |
| EU | 2000-2007 | -2.40 * | 2007-2013 | -0.59 | 2013-2020 | -2.71 * |  |  |  |  | -2,25 |
| Canada | 2000-2022 | -2.05 * |  |  |  |  |  |  |  |  | -2,05 |
| USA | 2000-2022 | -1.80 * |  |  |  |  |  |  |  |  | -1,80 |
| Argentina | 2000-2017 | 0.90 * | 2017-2022 | -2.01 |  |  |  |  |  |  | -0,73 |
| Brazil | 2000-2004 | -18.87 * | 2004-2021 | 0.85 |  |  |  |  |  |  | 0,85 |
| Chile | 2000-2021 | 0.77 * |  |  |  |  |  |  |  |  | 0,77 |
| Mexico | 2000-2022 | -0.01 |  |  |  |  |  |  |  |  | -0,01 |
| Australia | 2000-2023 | -2.89 * |  |  |  |  |  |  |  |  | -2,89 |
| Japan | 2000-2021 | -1.48 * |  |  |  |  |  |  |  |  | -1,48 |
| Republic of Korea | 2000-2022 | -1.59 * |  |  |  |  |  |  |  |  | -1,59 |

APC, annual percent change; AAPC, average annual percent change.
